# Genetic complexity at expression quantitative trait loci

**DOI:** 10.1186/s12919-016-0010-4

**Published:** 2016-10-18

**Authors:** Rita M. Cantor, Calvin Pan, Kimberly Siegmund

**Affiliations:** 1 Department of Human Genetics, David Geffen School of Medicine at UCLA, 695 Charles E. Young Dr, South, Los Angeles, CA 90024-7088 USA; 2 Department of Preventive Medicine, Keck School of Medicine of USC, SSB 202WHealth Sciences Campus, Los Angeles, CA 90089-9234 USA

## Abstract

**Background:**

Identifying variants that regulate gene expression and delineating their genetic architecture is a critical next step in our endeavors to better understand the genetic etiology of complex diseases. The appropriate genomic tools are in place, and preliminary analytic strategies have been developed.

**Methods:**

Here we used Genetic Analysis Workshop (GAW) 19 data to investigate the genetic complexity of expression quantitative trait loci (eQTL), chromosomal regions likely to harbor regulatory elements responsible for gene expression. For this investigation, we analyzed the lymphocyte expression profiles of 653 individuals in 20 pedigrees who were also genotyped by single nucleotide polymorphism (SNP) arrays, followed by sequencing and imputation. We used these data to examine the degree of allelic heterogeneity, a contributor to genetic complexity at eQTL, by sequentially conditioning on the most significantly associated SNPs.

**Result:**

SOLAR (Sequential Oligogenic Linkage Analysis Routines)-MGA (measured genotype approach) and FaST-LMM (Factored Spectrally Transformed Linear Mixed Model) software allowed us to analyze pedigree data. The power and Type 1 error rates for single SNP association testing and multiple SNP sequential association testing were consistent for these programs. Sequential conditioning of the real expression data revealed substantial levels of allelic heterogeneity at the 2 eQTL examined, illustrating this feature of genetic complexity.

**Conclusions:**

eQTL exhibit substantial genetic complexity among and within pedigrees.

## Background

Genetic complexity derives from factors that contribute to the non-Mendelian inheritance of a trait. These contributors include polygenic inheritance, locus and allelic heterogeneity, mitochondrial inheritance, and gene–gene and gene–environment interactions. The data provided by Genetic Analysis Workshop (GAW) 19 [1] allowed us to conduct a study to delineate the genetic architecture of expression quantitative trait loci (eQTL) and assess evidence for allelic heterogeneity. A previous manuscript analyzing a larger sample of the data derived from the San Antonio Family Heart Study reports that 85 % of lymphocyte expression levels were significantly heritable, making them appropriate candidate traits for eQTL analyses in the GAW19 pedigrees. In the manuscript [2], heritability varied substantially among the transcript levels, and the median was 22.5 %. In the published analysis, eQTL were identified by mapping the transcript levels using the SOLAR (Sequential Oligogenic Linkage Analysis Routines) software [3] to conduct linkage analyses. Here, we focus on the *cis* transcripts that exhibited substantial logarithm of odds (LOD) scores that were as high as 32, with a locus-specific heritability of approximately 80 %. We examined these eQTL because we thought it would be easier to detect multiple association signals with small effects and/or single signals with large effects when the LOD scores are larger. However, such LOD scores would also be observed if each pedigree had its own transcript-driving highly penetrant allele for the expressed gene. Other, more complex scenarios, involving multiple independent transcript-driving alleles that are shared by the pedigrees would also lead to such strong evidence for linkage of transcript levels to gene regions. GAW19 expression and sequence data on the same individuals within pedigrees provide an excellent opportunity to explore the genetic architecture of eQTL, and we focused on 2 of the eQTL with the highest LOD scores, chosen for their different gene lengths [2].

We were enthusiastic about the opportunity provided by GAW19 to assess the genetic architecture of eQTL using pedigrees, where the standard analytic approach of linear regression was not directly applicable. We compared 2 approaches appropriate for pedigrees, and used the GAW19 simulated data, where the models of inheritance employed to generate the data were known, in order to assess the power and Type 1 errors to detect associations for both single and multiple independent single-nucleotide polymorphisms (SNPs). These were the measured genotype approach as programmed in SOLAR [3] and the linear mixed-model approach as programmed in FaST-LMM (Factored Spectrally Transformed Linear Mixed Model) [4, 5]. Both programs were also used to investigate the genetic architecture of associated eQTL variants by employing the real data, where the models of inheritance are unknown.

## Methods

### Testing association in pedigrees: SOLAR-MGA and FaST-LMM

The variance component approach to gene mapping in pedigrees involves modeling a vector of pedigree member trait value deviations from the pedigree mean and a covariance matrix of kinship coefficients among the pedigree members. Given the appropriate constellation of relatives, the covariance matrix can be partitioned into additive genetic effects, along with the effects of genetic dominance and a common environment. In SOLAR-MGA [3], the genotypes to be tested for association are modeled by parameters in the individual trait values, and the tests are conducted using nested models and likelihood ratios. To reduce computation time and complexity, SOLAR-MGA applies eigen simplification of the likelihood function, where a vector of nonindependent observations is transformed to a vector where the observations are independent [6]. This reduces the likelihood function to the product of univariate normal densities, and the decomposition of the covariance matrix is composed of diagonal matrices of phenotypic and additive genetic eigenvalues. FaST-LMM is designed to perform genome-wide association studies (GWAS) when the relationships among the individuals in the study sample are unknown [4]. Linear mixed models capture these relationships and transformation of the estimated matrix of pairwise relationships is used to speed the analysis. Carefully chosen GWAS SNPs genotyped on the study sample are used to estimate genetic similarity. This estimation is done using SNPs from all chromosomes except the single chromosome containing the locus being tested for association [5].

### Estimating power and Type 1 errors for SOLAR-MGA and FaST-LMM

To assess power and Type 1 errors for detecting the individual associated SNPs using both analytic approaches, SOLAR-MGA and FaST-LMM, the simulated GAW19 pedigree data [1] in 200 replicates for the 6 associated SNPs in the *MAP4* gene on chromosome 3 were analyzed. For each replicate, taken separately, those on medication were removed from the analyses, and the diastolic blood pressure (DBP) and systolic blood pressure (SBP) quantitative traits were adjusted for age and sex. In the 200 simulated data sets, an average of 765 people, with a range of 742 − 783, remained in the study sample. For DBP, the age covariate had a mean effect of 0.07 with a range of 0.008 − 0.12 and the sex covariate had a mean effect of -3.15 with a range of -5.2 to -1.7 over the 200 replicates. For SBP, the age covariate had a mean effect of 0.45 with a range of 0.38 − 0.52, and the sex covariate had an effect mean of -4.6 with a range of -7.6 to -2.0. Power was assessed using the 0.05 and 5e-8 levels of significance in each simulated sample of 200. The Type 1 error was estimated using the 200 replicates of simulated trait Q1, which was not associated with the SNPs in *MAP4*. Power and Type 1 errors were also estimated for the sequential analysis approach that was subsequently applied to the real data. As with the single SNP assessments, simulated Q1, DBP, and SBP traits were used in each of the 200 replicates. To assess power, the analyses were conditioned sequentially on the *MAP4* SNPs.

### Assessing the genetic architecture of TIMM10 and LR8 eQTL

Two genes were selected randomly according to length (one shorter than the mean of the genes and one longer than that mean) among those with the 10 highest eQTL LOD scores in this pedigree cohort [2] for association and genetic architecture studies. Table 1 gives the names, IDs for the molecular probes, LOD scores, and base pair (bp) ranges for these genes, where 5000 bps have been appended to both sides of each gene. The genotyped SNPs, sequence data and imputed sequence data in these pedigrees has been used to identify the SNPs with genotypes within these 2 regions. Table 1 also provides the numbers of SNPs tested within each region. SOLAR-MGA and FaST-LMM were used to identify the independent signals among the 47 and 180 SNPs tested within these 2 genomic regions. Using a *p* value of 5e-8 and a *p* value of 0.05 to view the full range of possibilities, the conditioning options of SOLAR-MGA and FaST-LMM were used in an iterative fashion, conditioning on the single most statistically significant signal from the set, to generate the number of independent signals in each region, and this was done sequentially until no significant SNPs were observed in the analysis.

**Table 1:** Independent eQTL SNP associations by sequential conditioning using *p* <5e-8

Gene name	Probe_id	LOD	Base pair range	# SNPs tested	# of SNPs conditioned	# Significant SNPs	Minimum *p* value
TIMM10	GI_6912707-S	37	12120	47	0 1	8 9	2e-66 9.9e-86
LR8	GI_21361500-S	43	19100	180	0 1 2	29 14 1	9e-83 2e-22 4e-12

## Results

Table 2 provides estimates of the power of SOLAR-MGA and FaST-LMM to detect associated SNPs with locus-specific variances ranging between 0.028 and 0.002 in simulated traits for 2 levels of significance. The results generated by the 2 programs for these simulated data are remarkably consistent. The top 2 SNPs consistently show very strong power and the bottom 2 SNPs lack adequate power regardless of the level of significance used. Power for the 2 SNPs in the middle of the list shows substantial variation, depending upon the level of significance used. Three factors in the simulation model, given in column 2 of Table 2, contribute to this variation in power, which is positively correlated with the percent of variance explained, the minor allele frequency, and the absolute value of the effect. The Type 1 error was estimated to be 0.0475 for SOLAR-MGA and 0.07 for FaST-LMM when the level of significance was set at 0.05 for the trait Q1 that was not associated with any SNPs in the trait generating model.

**Table 2:** Power to detect single SNP associations using SOLAR-MGA and FaST-LMM, for *MAP4* simulated GAW19 pedigree data

Simulated diastolic blood pressure					
Chr–Bp	Prop. of variance explained/MAF/beta	SOLAR MGA power		FaST-LMM power	
		Alpha = 0.05	Alpha = 5 × 10^−8^	Alpha = 0.05	Alpha = 5 × 10^−8^
3 – 48040283	0.023/.03/-6.2	1	0.99	1	0.99
3 – 47957996	0.012/.03/-4.6	1	0.99	1	0.98
3 – 47956424	0.012/.37/-1.5	0.99	0.04	0.99	0.02
3 – 48040284	0.009/.01/-7.0	0.69	0	0.58	0
3 – 47913455	0.004/.005/-5.5	0.52	0	0.58	0
3 – 47957741	0.002/.002/-5.1	0.13	0	0.06	0
Simulated systolic blood pressure					
Chr–Bp	Proportion of variance Explained/MAF/beta	SOLAR-MGA power		FaST-LMM power	
		Alpha = 0.05	Alpha = 5 × 10^-8^	Alpha = 0.05	Alpha = 5 × 10^-8^
3 – 48040283	0.028/.03/-9.9	1	0.99	1	0.99
3 – 47957996	0.015/.03/-7.4	1	0.99	1	0.99
3 – 47956424	0.014/.37/-2.4	0.99	0.03	0.98	0.01
3 – 48040284	0.011/.01/-11.1	0.77	0	0.66	0
3 – 47913455	0.004/.005/-8.7	0.48	0	0.56	0
3 – 47957741	0.003/.002/-8.1	0.12	0	0.09	0

*Chr* chromosome, *Bp* base pair, *MAF* minor allele frequency

Table 3 reports the power analysis of the simulated DBP data, when conditioning sequentially on the *MAP4* SNPs that have been modeled to be associated with SBP and DBP. Both programs are consistent, so we only provide the information once. The first row gives the number of *MAP4* SNPs upon which the likelihood function has been conditioned sequentially. Rows 2 and 3 give the power to observe exactly that number of SNPs, sequentially, and the power to observe that number of SNPs and greater, sequentially, for *p* <0.05. The last 2 rows give the same for *p* <5e-8. The power for the 0.05 level of significance is appropriate, but the sample is substantially underpowered to provide adequate power for the simulated effect sizes. Estimated Type 1 errors for significance thresholds of 0.05, 0.01, and 0.001 are consistent with what is expected for both programs.

**Table 3:** Power to detect multiple SNP associations using sequential analyses for *MAP4* simulated GAW 19 pedigree data

# of SNPs detected		0	1	2	3	4	5	6
*P* <0.05	Exactly n	0	0	0	.05	0.21	0.62	0.13
	≥ n	0.999	0.999	0.999	0.999	0.95	0.74	
*P* <5e-8	Exactly n	0.01	0.01	0.945	0.035	0	0	0
	≥ n	0.999	0.99	0.98	0.035	0	0	

Table 1 reports the numbers of single associations and the conditioned results using the very stringent 5e-8 level of significance for TIMM10 and LR8. Both programs are consistent at this level of significance, and only the SOLAR-MGA analysis is reported. Introductory information regarding the genes is given in columns 1 to 5 and the last 3 columns report each step in the sequential analysis by giving the number of SNPs conditioned on at that step, the number of SNPs that are significant at that step, and the minimum *p* value among the significant SNPs at that step. Table 4 reports results analogous to those in Table 1 using a 0.05 level of significance. Once more, results of the 2 programs are consistent. These conditioned analyses support the existence of a large number of independent association signals for these 2 eQTL. The correct level of significance is not straightforward, as this is a multistep process. The most salient inference one can draw is that even at this stringent level of significance, there is evidence of multiple, independent, strongly associated signals, supporting eQTL complexity resulting from allelic heterogeneity. Additional analyses to investigate the genetic complexity of eQTL within pedigrees reveal patterns as complex as those seen in Table 4 (data not shown). Very few pedigrees only show a single associated SNP.

**Table 4:** Independent eQTL SNP associations by sequential conditioning using *p* <0.05

Gene		Enumeration of independent signals by association software			
		FaST-LMM		SOLAR-MGA	
	# SNPs conditioned on	# Significant SNPs	Minimum *p* value	# Significant SNPs	Minimum *p* value
TIMM10	0	25	3e-68	24	2e-66
	1	23	2e-87	23	9.9e-86
	2	10	5e-07	10	2e-07
	3	2	0.03	1	0.05
	4	1	0.04	–	–
LR8	0	67	4e-86	65	9e-83
	1	39	2e-24	55	2e-22
	2	47	1-11	63	4e-12
	3	46	0.0001	41	0.0002
	4	37	0.0001	39	0.0001
	5	40	0.0003	41	0.0002
	6	23	0.0004	22	0.0001
	7	14	0.00002	17	0.005
	8	14	0.003	8	0.01
	9*	8	0.002	8	0.02

*Six additional independent signals using SOLAR-MGA (0.019 < *p* <0.05)

## Discussion

Association testing of SNPs, conditioning sequentially with SOLAR-MGA and FaST-LMM, identified multiple independent SNP signals within each of 2 eQTL regions exhibiting high LOD scores in the GAW19 pedigrees. These analyses provide clear evidence that the genetic architecture of at least some eQTL exhibit allelic heterogeneity, with multiple independent signals, and, thus, are complex. Both software packages provide consistent power and Type 1 errors when testing for association with and without conditioning in the simulated data. The inference of complexity and multiple independent *cis-*regulatory elements is supported by a functional analysis of the F7 gene in an independent study [7].

## Conclusions

We investigated the presence of allelic association at eQTL in the GAW19 real expression data by conducting analyses that condition sequentially on associated SNPs in the full sample of pedigrees and within individual families. These analyses lead us to conclude that there are multiple independent eQTL for individual expression levels at the same locus. We are able to conclude that eQTL are genetically complex both across multiple pedigrees and within individual pedigrees. In addition, when comparing software that can be used for these analyses, statistical Type 1 error and power assessment on the simulated data GAW19 indicates that the results of FaST-LMM are consistent with those of SOLAR-MGA.

## References

[1] Blangero J, Teslovich TM, Sim X, Almeida MA, Jun G, Dyer TD, Johnson M, Peralta JM, Manning A, Wood AR (2015). Omics-squared: Human genomic, transcriptomic and phenotypic data for Genetic Analysis Workshop 19. BMC Proc.

[2] Göring HH, Curran JE, Johnson MP, Dyer TD, Charlesworth J, Cole SA, Jowett JB, Abraham LJ, Rainwater DL, Comuzzie AG (2007). Discovery of expression QTLs using large-scale transcriptional profiling in human lymphocytes. Nat Genet.

[3] Almasy L, Blangero J (1998). Multipoint quantitative-trait linkage analysis in general pedigrees. Am J Hum Genet.

[4] Lippert C, Listgarten J, Liu Y, Kadie CM, Davidson RI, Heckerman D (2011). FaST linear mixed models for genome-wide association studies. Nat Methods.

[5] Listgarten J, Lippert C, Kadie CM, Davidson RI, Eskin E, Heckerman D (2012). Improved linear mixed models for genome-wide association studies. Nat Methods.

[6] Blangero J, Diego VP, Dyer TD, Almeida M, Peralta J, Kent JW, Williams JT, Almasy L, Göring HH (2013). A kernel of truth: statistical advances in polygenic variance component models for complex human pedigrees. Adv Genet.

[7] Sabater-Lleal M, Chillón M, Howard TE, Gil E, Almasy L, Blangero J, Fontcuberta J, Soria JM (2007). Functional analysis of the genetic variability in the F7 gene promoter. Atherosclerosis.

